# Autologous retina transplantation for refractory highly myopic macular holes: a long-term follow-up

**DOI:** 10.1007/s10384-025-01169-4

**Published:** 2025-02-28

**Authors:** Matteo Mario Carlà, Carlos Mateo

**Affiliations:** 1 Ophthalmology Department, “Fondazione Policlinico Universitario A. Gemelli, IRCCS”, Largo A. Gemelli, 8, 00168 Rome, Italy; 2 Ophthalmology Department, Catholic University “Sacro Cuore”, Rome, Italy; 3 Instituto de Microcirugía Ocular (IMO), Barcelona, Spain

**Keywords:** Highly myopic macular hole, Myopic traction maculopathy, Autologous retinal transplantation, Refractory macular hole, Cystoid macular edema, Retinal graft

## Abstract

**Purpose:**

To evaluate long-term anatomical and functional outcomes of autologous retinal transplantation (ART) in refractory highly myopic macular holes (HMMHs).

**Study design:**

Retrospective interventional analysis of 9 eyes with refractory HMMH undergoing ART.

**Methods:**

Best-corrected visual acuity (BCVA, Snellen) and optical coherence tomography (OCT) were performed at baseline and each follow-up visit (1, 3, 6, 12, 24 months and the most recent). Preoperatively, we collected minimum linear diameter (MLD) and basal diameter (BD). Post-operatively, central macular thickness (CMT), external limiting membrane (ELM)/ellipsoid zone (EZ) visibility, macular edema (ME) and retinal pigmented epithelium (RPE) atrophy were evaluated.

**Results:**

Mean follow-up duration was 46.0 ± 19.6 months. Anatomical success was reached in 7/9 eyes (78%). Median BCVA went from 0.05 (IQR 0.065) at baseline to 0.075 (IQR 0.069) at final follow-up (*p* = 0.25). Only one eye showed a 2-lines improvement, while BCVA was stable in 4/9 (44%) and worsened in 1 eye (12%). CMT progressively thickened in the first 6 months (177 ± 68 μm), but then decreased to 122 ± 50 μm at final follow-up. Graft merging with the surrounding retina was visible in two eyes, showing partial ELM/EZ recovery and good outcomes. Microcystic-like refractory ME (33%) and long-term RPE atrophy (22%) were reported, while delayed displacement of the graft was seen in one case 6 months after first surgery.

**Conclusion:**

ART offered acceptable anatomical success but no visual improvement in our cohort. Lack of graft merging with the surrounding retina, persistent microcystic-like ME, RPE atrophy and hole recurrence were the most frequent shortfalls.

**Supplementary Information:**

The online version contains supplementary material available at 10.1007/s10384-025-01169-4.

## Introduction

Degenerative myopia is a major cause of legal blindness in developed countries [1]. The excessive elongation of the globe, causing posterior staphyloma, combined with the tangential traction caused by a tightened internal limiting membrane (ILM) and the anteroposterior traction due to the strong adhesion of the vitreous cortex, determine the spectrum of myopic traction maculopathy (MTM) [2, 3], including inner and outer retinal schisis, foveal detachment, and lamellar or full-thickness highly myopic macular holes (HMMHs) [4–6]. In particular, a combination of anteroposterior vitreomacular traction and tangential vector forces from the ILM lead to the formation of foveal cysts and disruption of outer retinal layers [7, 8]. In 8% of instances, the anteroposterior traction pressures might lead to the creation of HMMH and possible development of macular hole retinal detachment (MHRD) [9–11].

While emmetropic macular holes showed good results using pars plana vitrectomy (PPV) and ILM peeling together with gas tamponade, sealing HMMH is more difficult and has shown varying results [12]. The introduction of the inverted ILM flap method led to anatomical success rates of around 90%, even in cases with MHRD [13, 14]. However, the closure rates are lower in large HMMH, especially when adjusting for axial length (AXL), as recently proposed [15]. Around 5% of myopic patients develop a refractory HMMH after first surgery [16]. Treatment options for persistent HMMH after prior vitrectomy with ILM peel are restricted, including autologous transplantation of a free ILM flap [17], lens capsule transplantation [18] and, more recently, subretinal human amniotic membrane (hAM) patch [19, 20].

In 2016, Grewal et al. described the autologous retinal transplantation (ART) surgery for treating patients with refractory MHs [21], and this technique was validated in several successive investigations [22–25]. A multicenter case series investigation showed that the anatomic closure rate was 87.8% in patients undergoing ART, including emmetropic and myopic eyes. Furthermore, optical coherence tomography (OCT) showed that recovery in the outer retina structure could be seen with this technique, leading to better visual acuity in 52.3% of eyes with a closed MH [26]. Recently, Li et al. applied the ART technique to patients with refractory MHRD and followed them for 3 months after silicone oil removal, showing an 80% success rate and good visual outcomes [27].

However, research focusing on long term effectiveness of ART technique in HMMHs still lacks. Starting from this assumption, the aim of this research was to assess functional and anatomical outcomes of PPV and ART in refractory HMMHs, followed for at least 2 years. Moreover, an OCT analysis was performed to analyze postoperative complications and evolution of the ART graft.

## Patients and methods

Retrospective mono-centric interventional analysis of patients undergoing PPV and ART for refractory HMMHs was performed at the Instituto de Microcirugia Ocular, Barcelona, Spain, between October 2016 and May 2021. Follow-ups were recorded until January 2024. This research adhered to the tenets of the Declaration of Helsinki and was approved by the Instituto de Microcirugia Ocular Ethical Committee. All included subjects signed an informed consent.

High myopia was defined as an AXL > 26.50 mm or refractive error > 6 diopters (D). The following criteria were used for inclusion: presence of recurrent or refractory HMMH with or without MHRD, surgical treatment with PPV and ART, and a minimum 24-month follow-up time. All eyes had already undergone surgical treatment with ILM peeling and experienced primary surgical failure (refractory HMMHs) or reopening of the hole during post-operative follow-up (recurrent HMMHs). Exclusion criteria were: concomitant myopic choroidal neovascularization (mCNV), amblyopia, severe glaucoma, active uveitis, optic neuritis, history of cataract surgery in the previous 12 months, history of Irvine-Gass syndrome and severe systemic disease affecting ocular health. From a total of 325 HMMHs, 9 eyes met the inclusion criteria (flow chart in Supplemental file 1).

At baseline and every follow-up visit, a thorough ophthalmological anamnesis was performed, along with complete ophthalmic examination after pupil dilation. Data regarding previous operations were collected. Examinations included best-corrected visual acuity (BCVA, Snellen equivalent), intraocular pressure (IOP) evaluation, spectral domain (SD)-OCT evaluation with the Cirrus 5000 high-definition OCT (Carl Zeiss) and ultra widefield fundus photography (Optomap, Optos). All patients underwent full subjective distance visual refraction using a Snellen optotype at each visit. Very low visual acuity (counting fingers, hand motion) was converted following previous reports [28]. Postoperative visual improvement was considered as an increase of at least two lines of vision at the Snellen chart.

Each patient underwent the following scan protocols: 6 × 6 mm Macular Cube, horizontal and vertical HD Raster 21-line and HD Cross. The OCT scans were repeated at least once to ensure the quality of every image. Axial length was also measured at the baseline (IOLMaster, Carl Zeiss Meditec). Follow-up examinations were scheduled at 1, 3, 6, 12, 18, 24 months after the operation and at the most recent visit. All patients completed at least 24 months of follow-up.

In the fovea-centered horizontal B-scan, baseline minimum linear diameter (MLD) and basal diameter (BD) of the HMMH were calculated using the in-built caliper tool. MLD was measured at the narrowest distance between the edges of the broken margins of the neuroepithelia with a line roughly parallel to the retinal pigmented epithelium (RPE). BD was defined as the length of the RPE with detached photoreceptors, and its calculation was waived in cases of MHRD. In the post-operative period, central macular thickness (CMT) and visibility of external limiting membrane (ELM) and ellipsoid zone (EZ) were evaluated at each follow-up. Moreover, the eventual presence of macular edema (ME) or morphologic changes to the ART graft were reported.

### Surgical procedure

The same skilled vitreoretinal surgeon (C.M.) performed all surgical procedures using a 23-gauge system (DORC Eva Nexus, DORC) under peribulbar anesthesia. At first, Brilliant Blue G was injected around the HMMH inside the arcades to verify previous ILM peeling. In cases of MHRD, perfluorocarbon liquid (PFCL) was used to flatten the posterior pole retina before completing the peeling. At this point, a neurosensory retina harvest location was chosen in the mid-periphery, usually above the supero-temporal arcade, as described by Grewal [26]. The harvest size was first set at around 2 disc diameters and encircled by endolaser barrage. The graft was then cut using horizontal curved scissors and adjusted based on the size of the HMMH, in order to reach around 1.5 times the diameter of the HMMH.

The graft was delicately positioned towards the hole, ensuring that the outer retina surface of the graft faced the foveal RPE and that the HMMH was totally covered. Residual subretinal fluid was removed from the peripheral retina incision site using a soft-tipped needle. Following fluid–air exchange and PFCL removal, a gas tamponade (20% sulfur hexafluoride, SF6) or silicone oil (SO, in cases of MHRD) were injected. All patients had to keep a prone posture for a minimum of one week after the surgery.

The main outcomes of this study included anatomical closure of HMMHs, BCVA evolution and assessment of retinal graft morphology. Post-operative complications such as vitreous hemorrhage, hole reopening, endophthalmitis, and IOP elevation were reported.

### Statistical analysis

Statistical analysis was performed using IBM SPSS software, version 27.0 (SPSS Inc). The Shapiro-Wilk test was employed to assess the normality of the sample. Multiple comparisons test with Dunnett’s correction was used for continuous variables’ comparison between baseline and postoperative data. Correlation analysis was performed using Spearman coefficient. Visual acuity was reported as median with interquartile range (IQR) and compared with the Wilcoxon signed rank test. *P* < 0.05 was deemed statistically significant.

## Results

Nine eyes of 9 patients were included in this study. Mean age at presentation was 63.8 ± 9.6 years and the male/female ratio was 3/6. Right/left eye ratio was 6/3, and mean number of previous HMMH surgical procedures was 2.0 ± 1.1. Mean symptoms duration (e.g. metamorphopsia, central scotoma, loss of vision) at presentation was 6.8 ± 6.7 months, while average AXL was 31.63 ± 1.99 mm. Mean follow-up duration was 46.0 ± 19.6 months (range 24–78). Mean MLD at baseline was 597 ± 323 μm. Four cases (44%) had a MHRD at presentation. Demographic, clinical and OCT characteristics of the patients are visible in Table 1.

**Table 1:** Clinical and OCT characteristics of patients with refractory HMMH undergoing ART

ID	Age (Years)	Gender	Laterality	Lens status	Spherical equivalent(Diopters)	AXL (mm)	Previous HMMH surgery	Symptoms duration(months)	Baseline BCVA (Snellen equivalent)	MLD (µm)	Corrected MLD (µm) for AXL	BD (µm)	Tamponade	HMMH closure	Follow-up duration (months)	Final BCVA (Snellen equivalent)	Complications
01	75	F	OS	Pseudophakic	−1.5	30.58	1	6	20/400	431	549	568	SF6	Yes	45	20/400	None
02	64	M	OD	Pseudophakic	−0.25	29.98	1	5	20/200	1215	1518	MHRD	SO	No	78	20/200	Early graft displacement
03	56	F	OD	Pseudophakic	−2.25	31.05	2	6	20/500	600	776	1158	SF6	No	23	20/800	Late graft displacement, CME
04	66	M	OD	Pseudophakic	0	29.27	3	1	20/2000*	827	1009	MHRD	SO	Yes	59	20/200	RPE atrophy
05	58	F	OD	Aphakic	−3.5	34.69	2	3	20/200	137	198	180	SF6	Yes	65	20/60	None
06	62	F	OS	Pseudophakic	−2	32.44	1	18	20/400	682	922	853	SF6	Yes	50	20/400	RPE atrophy
07	78	F	OS	Aphakic	−3	33.28	1	12	20/400	478	663	589	SF6	Yes	24	20/200	None
08	47	M	OD	Aphakic	−5.5	29.55	4	2	20/2000*	597	735	MHRD	SO	Yes	46	20/2000	CME
09	68	F	OD	Pseudophakic	0	33.81	3	5	20/20,000*	406	572	MHRD	SO	Yes	24	20/100	CME

*HMMH* highly myopic macular hole; *BCVA* best-corrected visual acuity; *OCT* optical coherence tomography; *MLD* minimum linear diameter; *BD* basal diameter; *MHRD* macular hole retinal detachment; *SF6* sulfur hexafluoride; *SO* 5000 cSt silicone oil; *CME* cystoid macular edema; *RPE* retinal pigmented epithelium

*Converted from counting fingers (CF) at 2 feet (20/2000) and hand motion (HM) at 2 feet (20/20000)

None of the patients suffered intraoperative complications. In patients with previous MHRD, SO was removed in all cases 6 months after first surgery without complications. None of the cases developed recurrence of RD after SO removal.

Overall, anatomical success was reached in 7/9 eyes (78%). In one case, a refractory HMMH was identifiable at 1-month follow-up due to displacement of the retinal plug, while one patient experienced late HMMH recurrence 6 months after surgery. In both cases, patients decided not to undergo further operations, since no changes in BCVA were reported.

Median BCVA in the entire cohort showed an improvement from 0.05 Snellen equivalent, (IQR 0.065) at baseline to 0.1 (IQR 0.13) at 6 months follow-up (*p* = 0.04). However, BCVA at 1-year and 2-year follow-ups showed no significant improvement compared to baseline (*p* = 0.12 and *p* = 0.25, respectively). Final BCVA was 0.075 Snellen (IQR 0.069), which did not significantly differ from baseline (*p* = 0.25). Overall, at the end of the study, only one eye (patient 05) showed a significant 2-lines improvement in visual acuity, while 3/9 eyes (33%) showed a one-line improvement, 4/9 eyes (44%) were stable and a BCVA worsening was reported in 1 eye (12%).

Subgroup analysis between eyes with and without MHRD highlighted no significant differences in terms of anatomical success rates, BCVA improvement and prevalence of post-operative complications (all *p* > 0.05).

Two patients (ID 01 and 03) showed patchy subfoveal RPE atrophy at baseline due to myopic atrophic maculopathy (MAM), which remained stable during the follow-up period. However, none of these patients showed improvement in VA at any post-operative timepoint.

**Fig. 1:**
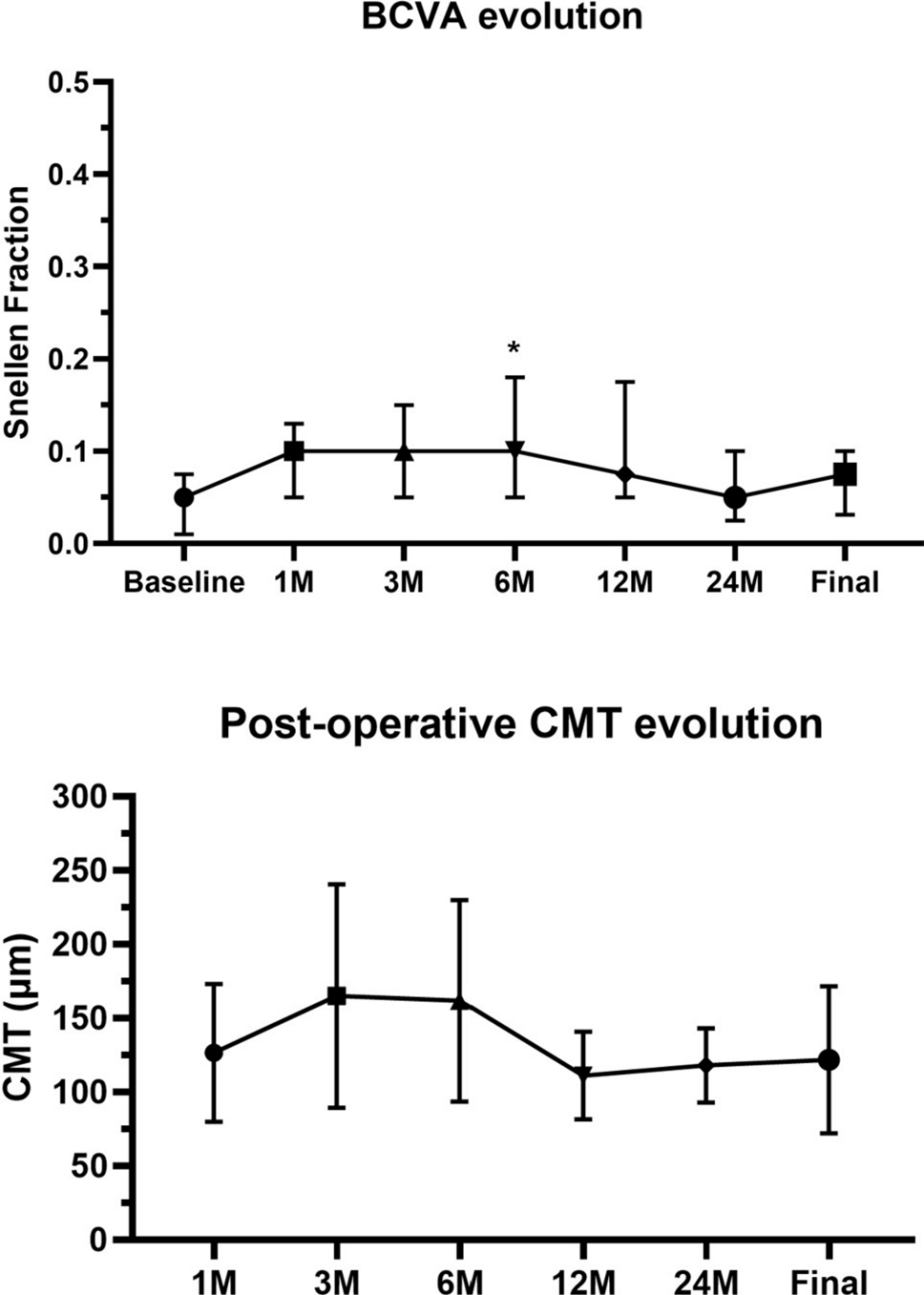
Evolution of best-corrected visual acuity (BCVA, upper graph) during follow-ups, expressed in median with interquartile range (IQR). After a significant improvement seen 6 months (6 M) after surgery, final BCVA did not significantly differ from baseline (T0). Similarly, central macular thickness (CMT, lower graph), measured in the post-operative period, showed a progressive thickening in the first 6 months, but then gradually decreased and stabilized from the 12 months follow-up

**Fig. 2:**
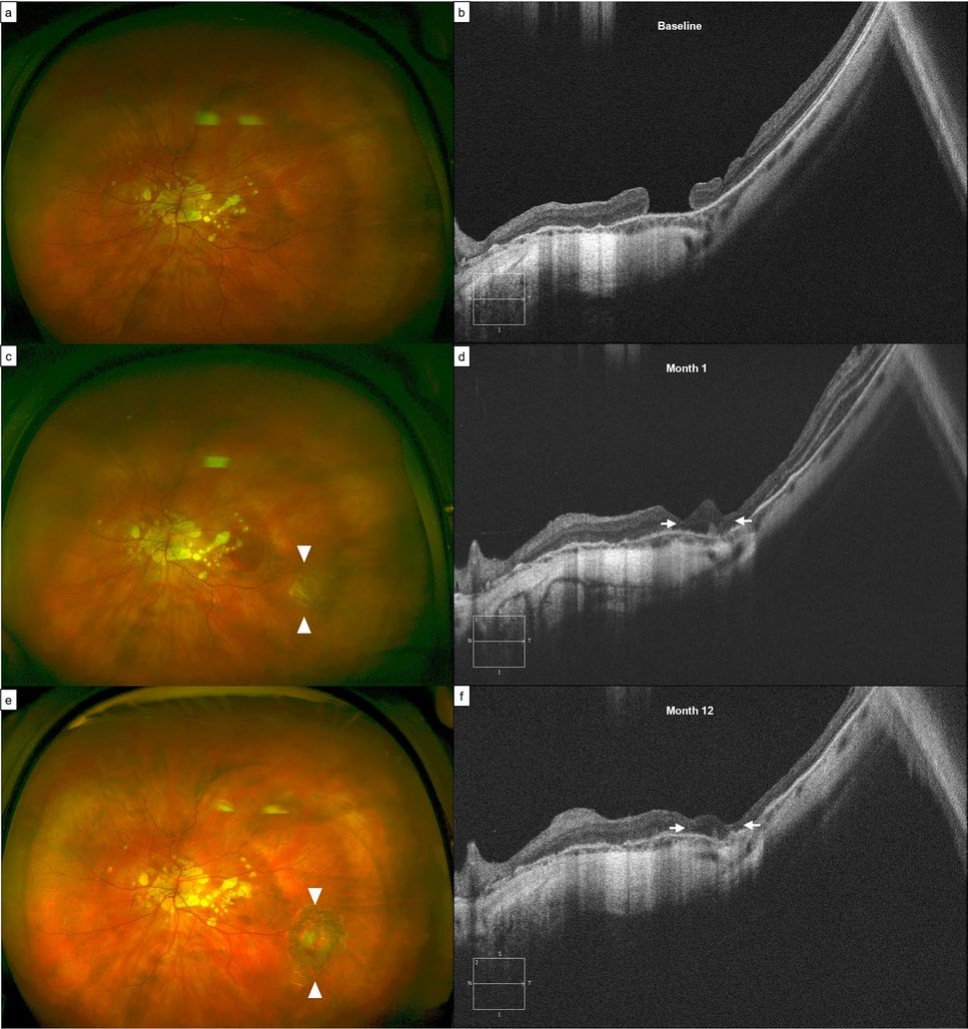
Case of a 56-years old woman (ID 01) undergoing autologous retinal transplantation (ART) for highly myopic macular hole (HMMH), seen at ultra-wide fundus photography (**a**) and B-scan (**b**). Post-operative fundus photography **c** and **e** shows the harvesting site of the retinal graft in the infero-temporal quadrant (white arrowheads), surrounded by 2–3 lines of laser barrage. Post-operative B-scans **d** and **f** show anatomical success with correct positioning of the retinal graft (white arrows), undergoing morphologic changes in the first post-operative year

**Fig. 3:**
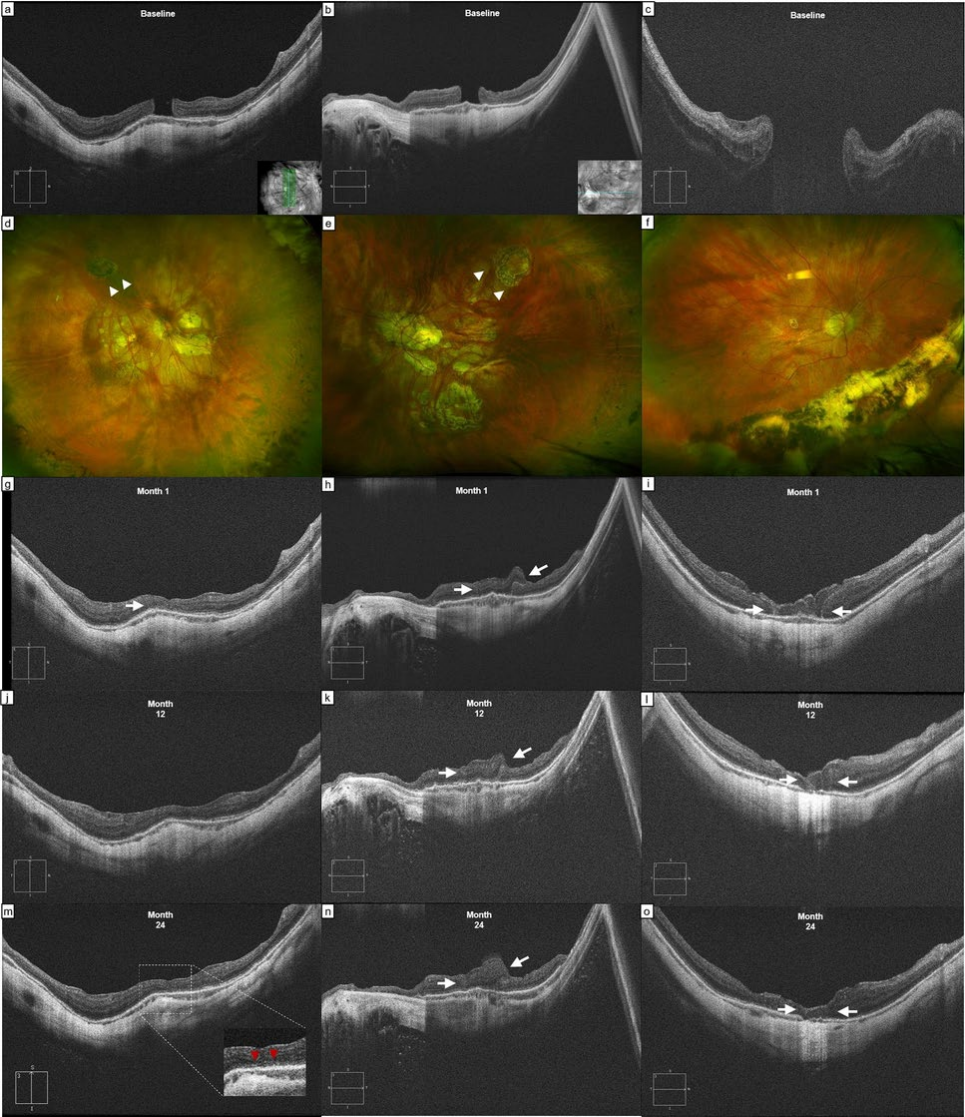
Cases of anatomical success after autologous retinal transplantation (ART) for highly myopic macular hole (HMMH). B-scans showing HMMHs (**a**, **b**), and concurrent macular hole retinal detachment (MHRD) in one case (**c**). Fundus photography, taken one month post-operatively (**d–f**), show the harvesting sites (**d**, **e**, white arrowheads). The optical coherence tomography (OCT) analysis showed a progressive merging of the graft in the first case (**g**, **j**, **m**), with partial reconstitution of a granular external limiting membrane (ELM)/ellipsoid zone (EZ) complex (red arrowheads), associated with good visual outcome. In the other two cases, the boundaries of the retinal graft were still visible 24 months after surgery (white arrows, images **h**, **k**, **n** and **i**, **l**, **o**, respectively) and the ELM/EZ complex was undetectable. Moreover, in the third case, a progressive sub-foveal atrophy, identifiable as a distinct area of choroidal hypertransmission, was visible after the first year of follow-up (**l**, **o**), and associated with poor visual outcomes

Postoperatively, we report a progressive thickening of CMT in the first 6 months, reaching 177 ± 68 μm, but then decreasing to 118 ± 25 μm and to 122 ± 50 μm at the 2-years and final follow-ups. (Fig. 1) In 6 eyes, the boundaries of the ART were still visible at the end of the follow-up. Two eyes (patient 05 and 09) showed apparent merging of the graft with the surrounding retina and continuous ELM and EZ lines. In any of the other eyes the ELM/EZ complex was detectable in the area of the previous HMMH at any follow-up (Figs. 2 and 3).

### Postoperative complications

None of the patients experienced postoperative vitreous hemorrhage or endophthalmitis.

**Fig. 4:**
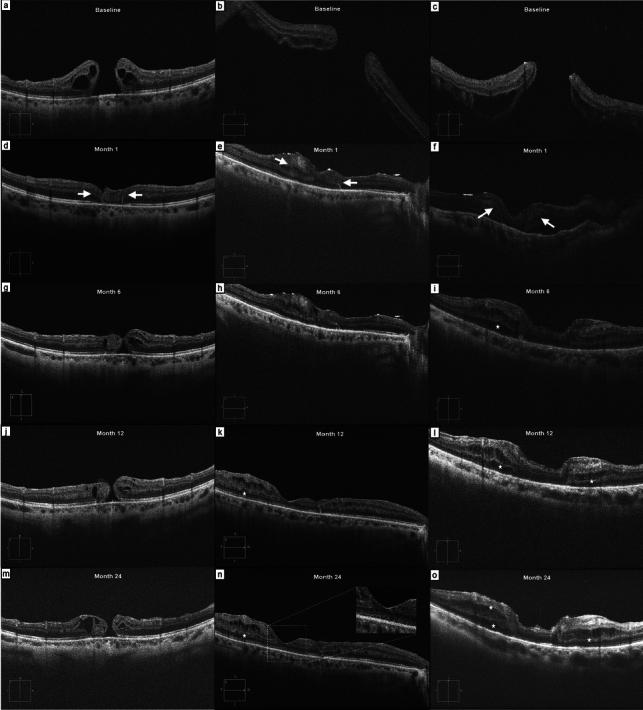
B-scans of three cases showing post-operative complications after autologous retinal transplantation (ART) for highly myopic macular hole (HMMH) and macular hole retinal detachments (MHRD) (**a–c**). One month after surgery, retinal graft was visible and successfully closed the hole (**d–f**). In the first case, the retinal graft showed a progressive sliding and cystoid degeneration, leading to HMMH recurrence at 6-months follow-up (**g**). During follow-ups, a worsening cystoid alteration of the graft and perifoveal retina was visible (**j**, **m**), but the patient decided not to undergo further surgery. In the other two cases, microcystic-like macular edema (ME) predominantly appeared in the inner nuclear layer (INL, white stars) and was refractory to intravitreal corticosteroids (**i**,** k**, **l**,** n**,** o**). In the second case, B-scan showed reconstitution of an apparently continuous external limiting membrane (ELM)/ellipsoid zone (EZ) complex, even if characterized by significant granularity, after 6, 12 and 24 months (**h**, **k, n**). In the third case, the boundaries of the graft were still visible after 24 months (**o**) and the visual outcome was poor

Overall, 3 eyes (one with HMMH and two with MHRD) developed post-operative chronic ME-like changes in the peri-foveal area (33% of the entire cohort). In one eye, the ME was highlighted starting from the 3-month follow-up, while the other 2 cases developed ME from the 6-months follow-up. The ME was localized in both the INL and ONL in 1 case, and only in the INL in 2 cases, showing a microcystic-like aspect. All three patients underwent ineffective intravitreal dexamethasone implant, and the ME was still visible 24 months after surgery (Fig. 4).

Two eyes (22%) developed progressive RPE atrophy in the area behind the retinal graft (one with HMMH and one with MHRD), with visible choroidal hypertransmission at the OCT one year after surgery, leading to poor visual outcomes. (Fig. 3).

In one case (patient 03), the retinal graft showed progressive contraction (from a 507 μm diameter at 1-month, to 347 μm and 265 μm at 6 and 24 months), cystoid degeneration and displacement toward the supero-temporal side, leading to HMMH recurrence 6 months after first surgery. In all other cases, the ART graft diameter remained stable over time (Fig. 4).

## Discussion

Highly myopic macular holes are still a challenge for vitreoretinal surgeons, and various modified surgical techniques based on the ‘scaffold theory’ have been proposed [17, 18, 25, 29]. In 2016, Grewal introduced the ART approach for refractory MHs, suggesting that this flap might work either as scaffold or as macular plug to help sealing the hole [21], reaching anatomical closure rates of around 90% in a successive multicenter study. Moreover, 52.3% of patients whose MH had closed had an improvement in vision [26]. More recently, a global study gathering 130 cases of ART showed an 89% rate of MH closure, reaching 95% of closure in MHRD. In this report, 43% of eyes had a visual improvement of more than 3 lines, mainly associated with EZ reconstitution [30]. Finally, in the CLOSE study group collecting 1135 eyes from 35 articles, ART showed an 87% closure rate in extra-large MHs (> 1000 μm) [31].

In our research on refractory HMMH, anatomical closure was achieved in 78% of cases. However, median BCVA was not improved at the end of the follow-up period: only one patient gained at least 2 Snellen chart lines. Specifically, median BCVA progressively improved in the first 6 postoperative months, but then re-decreased over time. However, since we focused on eyes with significantly high AXLs (mean 31.63 mm), the progression of myopic atrophy maculopathy (MAM) may have impacted on visual outcomes. Moreover, when correcting the dimension of the holes for the AXL using a previously published formula [15], the mean MLD was 771 μm (range 549–1518), indicating a class of patient with poorer anatomical and functional outcomes. In addition, four cases had HMMH-associated RD at presentation, suggesting a pre-existing significant damage to photoreceptor cells. In contrast with our research, in a cohort of highly myopic eyes with MHRD undergoing ART, Li et al. report better post-operative visual improvement. However, in some cases, visual recovery was hampered due to factors such as atrophy of the RPE and choroid and prolonged RD. Several previous studies suggest that, in these complex cases, a permanent harm to the photoreceptor cells has already taken place, regardless of retina reattachment [1, 32, 33]. Finally, even if PFCL was employed for graft harvesting and positioning over the hole, we didn’t use it as an adjuvant in the early post-operative period in order to optimize graft stability, as recently suggested by Moysidis et al. [30]. Previous research also highlights that SO hinders oxygen transfer between the retina and the anterior chamber after PPV [34], thus reducing oxygen diffusion to the transplant. On the other hand, PFCLs theoretically offer better oxygen diffusion and perfusion, which could be critical for the merging of the retinal graft and improve visual outcomes [30].

Previous research reports that ELM and EZ reconstituted after ART, suggesting an unidentified migratory process that may integrate the flap with the original retina tissue, since the boundaries between the graft and MH edge eventually vanished [26]. These results were successively confirmed in the investigations of Thomas et al. and Parolini et al. [35, 36]. Similarly, a recent report by Lumi et al. highlights that microperimetry was able to show retinal function in the peripheral area of the retinal graft, while multifocal electroretinography (mfERG) showed abnormal function of the central ring and normal function of the second ring [37]. By contrast, in HMMH, we highlighted that the margins of the retinal graft were still visible in 75% of eyes, even years after surgery. Only two eyes showed partial reconstitution of ELM and EZ at OCT, and these of the patients with the best visual prognosis. Our results are in line with those of Li et al., whose study focused on 10 cases of refractory MHRD, in which none of the eyes showed restoration of the ELM or EZ [27]. Wu et al. and Ding et al. propose that the retinal graft contributed to the retinal structure and facilitated the restoration of the outer retinal structure at the margin of the MH [25, 38]. Caporossi et al., employing a subretinal hAM patch in recurrent HMMHs, suggest a possible promotion of outer retinal layers regeneration, namely the ELM and EZ, leading to retinal development and differentiation [20].

On the other hand, it is well known that the nutrition and oxygen demand of photoreceptors are mainly met by the choroid [39]. Recent reports highlight that in MHs undergoing successful ART, angiogenesis and anastomosis were seen in up to 35% of the eyes, likely contributing to the survival of the transplanted retina [40]. Tabandeh shows similar findings even in giant recurrent MHs [41]. However, in eyes with pathologic myopia, choroidal impairment is reported to increase proportionality with AXL, with a critical flexion point around 27.26 mm [42]. Starting from this assumption, ART technique in HMMHs may be hindered by a significant pre-existing choroidal vascular deficit, either in terms of visual outcomes, or in terms of graft fusion with the surrounding retina. In fact, we reported two cases of long-term RPE atrophy behind the graft, suggesting that choroidal impairment may hinder the hole healing process, leading to poor integration of the graft and imbalanced homeostasis of the RPE.

In the multicenter study of Grewal et al., graft displacement was the most common intraoperative and early post-operative complication, either with SO or with gas tamponade [26]. Similarly, we highlighted one case of early graft displacement and refractory HMMH at 1-month follow-up. Wu et al. and Liu et al. suggest that using neurosensory retina and autologous blood together might serve as both a bonding agent to lower the chances of graft dislocation post-surgery and as a possible enhancer to speed up the healing process [23, 25]. Furthermore, previous research advises that, in order to facilitate the growth of glial cells by acting as a bridge and scaffold, the retinal graft size should be larger than the diameter of the MH, since the graft may be subjected to retraction over time [21, 30]. Moreover, in highly myopic eyes, in particular with MHRD, the actual dimension of the HMMH is difficult to assess intraoperatively. Consequently, we decided to keep the retinal graft around 1.5 times larger than the HMMH diameter. This allowed to provide sufficient covering by the graft tissue on the hole surface, consistent with what is suggested by previous research [27]. Nevertheless, we reported a case in which the retinal graft progressively contracted and developed cystoid degeneration, leading to re-opening of the hole. This finding suggests that the retinal graft is not fully integrated even months after surgery and may undergo displacement or delayed contraction.

In their first report, Grewal et al. highlighted late development of post-operative CME in 17% of cases, usually around 4–6 months after surgery [21]. In our case series, 3 patients (33%) developed perifoveal CME-like changes starting at month-3 or month-6 follow-ups. These alterations were refractory to dexamethasone therapy and were still visible years after surgery. This edema was characterized by small cysts, predominantly localized in the INL, showing similarities to the microcystic macular edema (MME), previously described in optic neuropathies but recently associated with diabetic retinopathy, epiretinal membranes and ILM peeling. This kind of edema seems to develop as a result of Müller cell dysfunction associated with ganglion cell damage, resulting in poor visual prognosis [43]. Our results are consistent with more recent research, in which post-operative inner retinal edema appeared in up to 44% of cases, being part of graft remodeling and generally not impacting visual functionality [30, 44–46]. The failure of RPE pumping system, the breakdown of the blood-retinal barrier, extracellular fluid accumulation, and intraocular inflammation have all been reported to contribute to post-operative CME development [44]. Moreover, in highly myopic eyes, we hypothesize that the presence of the retinal graft may progressively induce further dysfunction of perifoveal Müller cells, leading to the appearance of chronic intraretinal cysts and suggesting that the retinal graft alters the homeostasis of surrounding retinal tissue even months after surgery. In parallel, we observed an increase of graft thickness during the first 6 months post-operatively, followed by a reduction and stabilization of CMT 12 months after surgery. We suggest that the same mechanism giving light to peri-foveal edema, particularly relying on graft remodeling and intraocular inflammation, leads to the swelling of the graft in the first post-operative months, rather than turning back to initial values over time.

Our study has several limitations. First, the study was retrospective and lacked a control group. Second, the sample size was small and included patients with and without MHRD at baseline, thus including patients undergoing either gas or SO tamponade. Nevertheless, patients were followed for at least 24 months and SO had been removed in all cases within the sixth post-operative month. Moreover, the follow-up period was variable among patients and this may have influenced visual outcomes and development of post-operative complications. Finally, other data such as the extent of retinal detachment, the size of the HMMH and the chorioretinal atrophy, might have affected anatomic and visual prognosis, highlighting the need of further research.

In conclusion, we report the results of ART in a small cohort of refractory HMMHs followed for at least two years, highlighting that, even if anatomical outcomes were acceptable in terms of closure rate, visual acuity didn’t show a significant improvement. Moreover, myopic patients showed several adverse effects in the post-operative period, such as HMMH re-opening and microcystic edema like-changes, while only two cases showed effective merging of the graft with the surrounding retina. Overall, in this specific subclass of patients, retinal graft offers excessively variable results, suggesting that an ideal technique has yet to be discovered.

## Electronic supplementary material

Below is the link to the electronic supplementary material.

Flow diagram highlighting the selection criteria of this research. HMMH=high myopic macular hole; ART=autologous retinal transplantation Supplementary Material 1
