# Supplementary figures and images for: Autologous retina transplantation for refractory highly myopic macular holes: a long-term follow-up
Source: Jpn J Ophthalmol. 2025 Feb 28;69(2):259–67. doi: 10.1007/s10384-025-01169-4 (PMC12003452; doi:10.1007/s10384-025-01169-4)

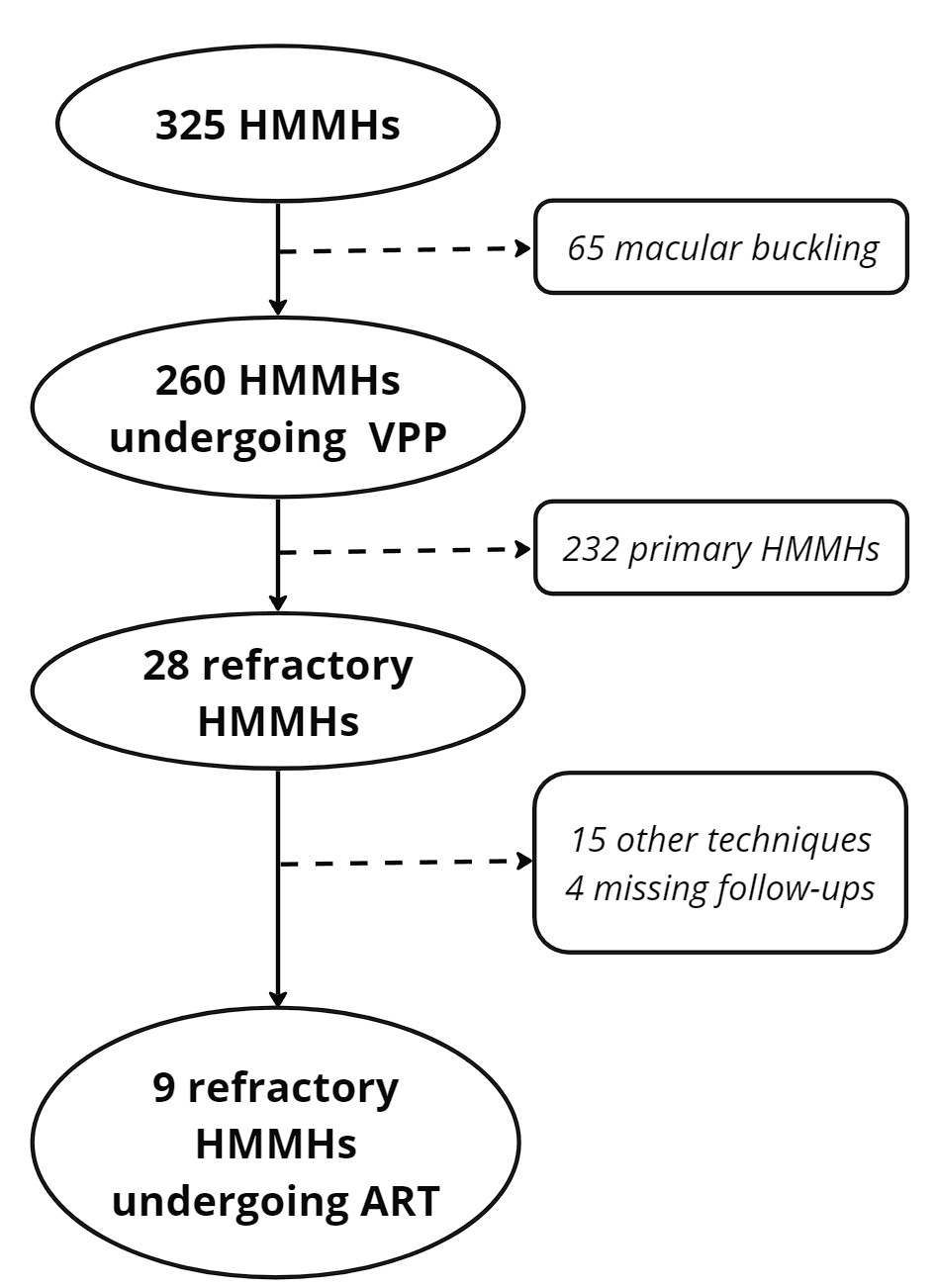

Supplement: Supplementary file 1 — Flow diagram highlighting the selection criteria of this research. HMMH=high myopic macular hole; ART=autologous retinal transplantation Supplementary Material 1 [file 10384_2025_1169_MOESM1_ESM.tiff]
